# Supercritical CO_2_ extraction of hemp seeds: A multivariate perspective on the influence of processing parameters on oil composition, antioxidant activity, and enzyme inhibition^☆^

**DOI:** 10.1016/j.fochx.2025.103296

**Published:** 2025-11-16

**Authors:** Prajakta Vishwasrao, Gokhan Zengin, Kouadio Ibrahime Sinan, Mirjana Minceva, Simon Vlad Luca

**Affiliations:** a Biothermodynamics, TUM School of Life Sciences, Technical University of Munich, 85354 Freising, Germany; b Physiology and Biochemistry Research Laboratory, Department of Biology, Science Faculty, Selcuk University, 42130 Konya, Turkey

**Keywords:** *Cannabis sativa*, Hemp seed oil, Supercritical fluid extraction, Fatty acids, Oil bioactivity, Tocopherols

## Abstract

Hemp seeds are valued for their unique nutritional and health benefits. This study examined the impact of supercritical (sc)CO_2_ extraction conditions on hemp seed oil yield, composition, antioxidant activity, and enzyme inhibition using a multivariate approach. While pressure (300–500 bar) had minimal effects, temperature (40–60 °C) and ethanol addition (0.6–1.5 %) significantly influenced oil yield. The levels of fatty acids, tocopherols, carotenoids, chlorophylls, phenolics, and flavonoids varied independently of extraction pressure and temperature, but their extractability generally increased with ethanol concentration. The co-solvent addition also enhanced radical scavenging activity but diminished the metal-reducing and chelating properties. Hemp seed oils inhibited enzymes linked to chronic diseases like diabetes, skin disorders, and Alzheimer's. Multivariate analysis grouped samples by fatty acid profile, pigment content, and bioactivity. This work provides novel insights into how scCO_2_ conditions affect the chemical and biological properties of hemp seed oils.

## Highlights

• Hemp seed oil was obtained by scCO2 extraction at different process conditions.
• The extraction of minor compounds from seeds was enhanced by ethanol addition.
• Ethanol addition improved the radical scavenging effects of obtained hemp oils.
• Hemp seed oils inhibited cholinesterases, tyrosinase, amylase, and glucosidase.
• Multivariate analysis differentiated hemp oils into chemical and bioactivity clusters.

## 1 Introduction

*Cannabis sativa* L. (hemp) seeds have been consumed as animal feed or for human nutrition for thousands of years. The whole hemp seeds are comprised of fiber, proteins, and contain more than 30 % oil.(Alonso-Esteban et al., 2020; Ostapczuk, Apori, Estrada, & Tian, 2021). Hemp seed oil is abundant in polyunsaturated fatty acids (PUFAs) and exhibits a favorable omega-6 to omega-3 fatty acid (ω6/ω3) ratio of 3:1 (Devi & Khanam, 2019b; Petrović, Debeljak, Kezić, & Džidara, 2015). This ratio is regarded as optimal in human nutrition, as it can significantly reduce the incidence of cardiovascular pathologies (Aiello et al., 2020; Siano et al., 2018; Vogl, Mölleken, Lissek-Wolf, Surböck, & Kobert, 2004). Hemp seed oil is typically produced by cold pressing or solvent extraction (e.g., Soxhlet). Despite its effectiveness, solvent extraction can leave harmful residues and pose environmental issues. Conversely, cold pressing, albeit favored for its gentle processing conditions, cost-effectiveness, and implementation simplicity, can extract only up to 65 % of the oil content from the seeds. (Aladić et al., 2015; N. Baldino et al., 2022). Thus, alternative technologies, such as supercritical fluid extraction, have been proposed to obtain high-quality hemp seed oil with enhanced yields (Aiello et al., 2020; Aladić et al., 2015; Aladić et al., 2014; N. Baldino et al., 2022; C Da Porto, Decorti, & Tubaro, 2012; Carla Da Porto, Natolino, & Decorti, 2015; C Da Porto, Voinovich, Decorti, & Natolino, 2012; Devi and Khanam, 2019a, Devi and Khanam, 2019b; Grijó, Piva, Osorio, & Cardozo-Filho, 2019; Kerner, Jõudu, Tänavots, & Venskutonis, 2021; Tomita et al., 2013). For example, the supercritical carbon dioxide (scCO_2_) extraction of hemp seeds was studied at temperatures ranging from 40 to 80 °C and pressures of 200 to 400 bar, with the maximum yield achieved at 80 °C and 400 bar (Tomita et al., 2013). Similar processing conditions (300–400 bar and 40–80 °C) were also documented by other groups following a series of optimization studies that utilized various experimental designs and statistical models (Aladić et al., 2015; C Da Porto, Decorti, & Tubaro, 2012; Carla Da Porto et al., 2015; C Da Porto, Voinovich, et al., 2012; Devi & Khanam, 2019b). In additional research, the quality of oils extracted using scCO_2_, evaluated through criteria such as oil stability, tocopherol levels, fatty acid composition, pigment content, and ω6/ω3 ratio, was compared to the quality of oils obtained through traditional extraction methods like Soxhlet and ultrasound-assisted extraction. The scCO_2_ oils were generally ranked as having a comparable or improved quality (Aladić et al., 2015; Aladić et al., 2014; N. Baldino et al., 2022; Devi & Khanam, 2019a). The use of liquid (subcritical, near-critical) CO_2_ or other supercritical solvents (e.g., propane, dimethyl ether) to extract hemp seed oil was also explored (Aiello et al., 2020; Ellison et al., 2021; Grijó et al., 2019).

In most earlier studies, with limited exceptions such as those cited by (Grijó et al., 2019), whole hemp seeds with their shells intact were utilized. Un-hulled hemp seeds typically contain 30–35 wt% oil. (Aiello et al., 2020; Alonso-Esteban et al., 2022). The de-hulling process, which involves the simple removal of the fiber-rich shell, can provide materials with an oil content of up to 55 wt% (Alonso-Esteban et al., 2020; Alonso-Esteban et al., 2022). Therefore, the low extraction yields noted in many of the previous works may be linked to using un-hulled seeds. In addition, most studies focus on total oil yield without presenting extraction kinetics curves, limiting the understanding of how processing conditions, like temperature, pressure, and co-solvent, could influence the oil yield dynamics (Carla Da Porto et al., 2015; Devi & Khanam, 2019a; Tomita et al., 2013). Furthermore, although oils extracted with scCO_2_ are known to possess a superior antioxidant profile compared to oils obtained with conventional methods (Aladić et al., 2014), their health-related effects have been poorly studied (Alonso-Esteban et al., 2022; Hong et al., 2015; Smeriglio et al., 2016; Yu, Zhou, & Parry, 2005).

The aim of this study was to investigate the impact of scCO_2_ extraction conditions on hemp seed oil yield, composition, antioxidant activity, and enzyme inhibition using a multivariate approach. In contrast to previous works that used whole un-hulled seeds with lower oil content, this study employed de-hulled seeds, allowing for higher oil content in the starting materials. As most studies report only total yields, lacking insight into extraction dynamics, the first objective of this work was to evaluate the influence of process conditions on scCO_2_ extraction by plotting extraction kinetics curves for all tested conditions (pressure, temperature, and co-solvent addition). The study's second goal was to examine the chemical composition of the oils, focusing on fatty acids, tocopherols, chlorophylls, carotenoids, total phenolics, and flavonoids. This approach provides a more comprehensive and detailed analysis than the literature, which tends to limit its scope to a narrower range of compounds. As prior research rarely links scCO_2_ extraction parameters to the biological activity of hemp seed oils, the third objective was to study the potential bioactivity of the oils obtained under different scCO_2_ extraction conditions, assessing antioxidant properties (reducing power, radical scavenging, chelating ability) and enzyme inhibition (cholinesterases, tyrosinase, glucosidase, and amylase). The final objective was to perform an exploratory multivariate analysis to evaluate the impact of different scCO_2_ extraction conditions on the chemical profile and bioactivity of the resulting oils. This study introduced a data-driven approach utilizing statistical tools such as principal component analysis, which is predominantly absent in preceding studies on scCO_2_ extraction of hemp seed.

## 2 Material and methods

### 2.1 Chemicals and materials

Hexane (≥95 %), isopropanol (≥95 %), acetonitrile (≥99 %), acetone (≥95 %), ethanol (≥99 %), hydrochloric acid (∼37 %), and methanol (≥99 %) were acquired from VWR Chemicals (Ismaning, Germany). Tocopherol standard mix, 2′-azino-bis(3-ethylbenzothiazoline-6-sulphonic acid (ABTS), tridecanoic acid (≥98 %), 1,1-diphenyl-2-picrylhydrazyl (DPPH), ammonium acetate, 2,4,6-tris(2-pyridyl)-*s*-triazine, ammonium molybdate, Folin-Ciocalteu reagent, butyrylthiocholine chloride, acarbose, porcine pancreas amylase (EC. 3.2.1.1), ferrozine, cupric chloride, ferrous sulfate hexahydrate, gallic acid, galanthamine, sodium hydroxide, sodium carbonate, fatty acid methyl ester (FAME) mix (C8-C24), ferric chloride, *Saccharomyces cerevisiae* glucosidase (EC. 3.2.1.20), sodium molybdate, horse serum butyrylcholinesterase (BChE, EC 3.1.1.8), eel acetylcholinesterase (AChE, type-VI-S, EC 3.1.1.7), kojic acid, mushroom tyrosinase (EC1.14.18.1) Trolox, acetylthiocholine iodide, ethylenediaminetetraacetate (EDTA), and sodium nitrate were bought from Merck (Darmstadt, Germany). CO_2_ (≥99.7 %) was from Westfalen AG (Münster, Germany).

The de-hulled hemp seeds (*Cannabis sativa* L. cv. Futura 75) were purchased from Hempro International (Düsseldorf, Germany). The seeds were ground with a grinder (Bosch, Gerlingen, Germany). The moisture content, assessed by placing a known amount of ground hemp seeds at 105 °C for 24 h and calculating the mass difference (3 repeated experiments), was 6.8 ± 0.7 wt%. The oil content, determined by Soxhlet extraction with hexane (10 g ground hemp seeds with 200 mL hexane for 6 h; 3 repeated experiments), was 52.2 ± 1.2 wt%.

### 2.2 Supercritical CO_2_ experiments

The scCO_2_ experiments were conducted on a Spe-ed SFE Zoran extractor (Applied Separations, Allentown, PA, USA) that can operate below 690 bar and 240 °C (Fig. S1). To carry out co-solvent experiments, the extractor was additionally connected to an external HPLC pump (KNAUER, Berlin, Germany). A stainless steel extraction vessel with a volume of ∼23 mL (1.4 cm internal diameter; 15 cm length) was used. In each experiment, the vessel was loaded with ground hemp seeds (5 g) and filled with glass beads (5 mm diameter) and a layer of glass wool at the bottom and top of the cell. The temperature of the extraction vessel was regulated by an electric thermostatic heating mantle. The vessel was connected to the extraction unit. The pressure was adjusted to the target value, and the timer started (time point 0 min). The CO_2_ flow at the outlet was adjusted to 7 standard liters per minute (SLPM, corresponding to 0.215 g/s) with a micrometer valve and a flow meter (ALICAT Scientific, Tucson, AZ, USA). The extract was recovered in pre-weighed glass tubes at 20 min intervals throughout the 140 min extraction period. In the co-solvent (ethanol) experiments, the ethanol contained in the collected fractions was removed with a rotary vacuum concentrator (RVC 2–25 CDPlus, Martin Christ, Osterode am Harz, Germany). The extraction yield of oil in each of the collected fractions was determined as follows: yield (g_oil_/g_seeds_) = weight of extract in fraction (g) / weight of the initial hemp seed sample (g). At the end of the extraction, the fractions were dissolved in hexane and pooled together. The pooled hemp seed oil was then obtained by evaporating the hexane at reduced pressure (<200 mbar) using a rotary evaporator (Heidolph Instruments, Schwabach, Germany). All scCO_2_ experiments were performed in duplicate.

### 2.3 Phytochemical analysis

#### 2.3.1 Fatty acid analysis

The fatty acid profile of the hemp seed oil samples was analyzed by gas chromatography–mass spectrometry (GC–MS), using the FAME method. Briefly, the oil sample (20 mg) was dissolved in hexane (1 mL). The hexane solution (200 μL) was pipetted into a vial, followed by the addition of methanol/hydrochloric acid (11/1; 6 mL) and 1 mg/mL tridecanoic acid (0.1 mL) as internal standard. The vial was placed in a thermostated block for 1 h at 90 °C. Subsequently, hexane (1.7 mL) and water (2 mL) were added, and the vial was shaken. After phase separation, the upper organic layer (1 μL) was analyzed by the GC (Agilent 6890 N, Agilent Technologies, Palo Alto, CA, USA). A Restek Rtx-Wax column (30 m × 0.25 mm i.d., 0.5 μm film thickness) was used. The flow rate of the carrier gas (helium) was 1 mL/min. The split ratio was set to 7.5:1, while the injector inlet was maintained at 200 °C. The oven was initially set at 80 °C for 2 min, then increased to 180 °C at 7 °C/min, where it was held for 10 min. The following MS parameters were applied: full scan mode, 50–350 amu, ionization energy of 70 eV, and MS source temperature of 230 °C. Fatty acid content (reported at.% total fatty acids) was quantified using a calibration curve prepared with a FAME reference mixture (C8-C24).

#### 2.3.2 Chlorophyll and carotenoid analysis

Chlorophylls and carotenoids were quantified with a previously reported method (Aladić et al., 2014). Briefly, the sample (200 mg) was dissolved in acetone (10 mL). After ultrasonication for 10 min and homogenization in a vortex mixer for 15 s, the absorbance was recorded at 470, 642.5, and 660 nm with a SPECORD 50Plus spectrophotometer (Analytik Jena, Jena, Germany). The total pigment content was calculated using the formulas provided in a previous publication (Aladić et al., 2014), with the data expressed in mg/kg oil.

#### 2.3.3 Tocopherol analysis

Tocopherols were quantified with a previously developed method (Aiello et al., 2020). Briefly, the sample (0.1 mL) was dispersed in isopropanol (1.9 mL). After homogenization in a vortex mixer (MS2 Minishaker, IKA, Staufen, Germany) for 15 s and filtration through syringe filters, 20 μL were injected into the HPLC. The HPLC system (Shimadzu, Tokyo, Japan) included a DGU-20 A3 degasser, LC-20 AB binary pump, SIL-20A auto-sampler, and SPD-M20A UV/VIS detector. A Capcell Pak C18 (250 mm × 4.6 mm i.d., 5 μm) column and 100 % methanol (mobile phase) were used. The flow rate was 0.8 mL/min, with the UV chromatograms processed at 292 nm. A calibration curve with the tocopherol mix (containing 56 mg/g α-tocopherol, 563 mg/g γ-tocopherol, and 108 mg/g δ-tocopherol) was constructed and used for expressing the content of the three tocopherol isomers in the hemp seed oils (mg/kg oil).

#### 2.3.4 Total phenolic and flavonoid content

Phenols and flavonoids were first extracted from the oil samples as follows: hemp seed oil (1 g) and methanol (3 mL) were shaken for 2 h in a shaker (Multi RS-60, Biosan, Riga, Latvia); the methanol-rich phase (1.5 mL) was evaporated in the rotary vacuum concentrator. The methanol extracts were then evaluated regarding the total flavonoid and phenolic content as described previously (Grochowski et al., 2017). The data were calculated as mg rutin equivalents (RE)/kg oil and mg gallic acid equivalents (GAE)/kg oil for the total flavonoid and phenolic content, respectively.

### 2.4 Biological activity evaluation

#### 2.4.1 Antioxidant assays

The antioxidant potential of hemp seed oils was assessed as previously detailed (Grochowski et al., 2017). The data were expressed as mg Trolox equivalents (TE)/100 g oil in the DPPH and ABTS radical scavenging assay, ferric ion reducing antioxidant power (FRAP), and cupric ion reducing antioxidant capacity (CUPRAC). For the metal chelating ability (MCA), the data were provided in mg EDTA equivalents (EDTAE)/100 g oil. For the phosphomolybdenum (PBD) assay, the results were calculated in mmol TE/100 g oil.

#### 2.4.2 Enzyme inhibition assays

The enzyme inhibition of hemp seed oils was assessed by targeting cholinesterases, tyrosinase, amylase, and glucosidase, as previously detailed (Grochowski et al., 2017). In the AChE and BChE assays, the data were expressed as mg galanthamine equivalents (GALAE)/100 g oil. In the tyrosinase assay, the outcomes were provided as mg kojic acid equivalents (KAE)/100 g oil, whereas the values were presented as mmol acarbose equivalents (ACAE)/100 g oil in the glucosidase and amylase assays.

### 2.5 Statistical and multivariate analysis

The analyses were conducted in triplicate, with the data reported as average ± standard deviation. One-way ANOVA with Tukey's post-hoc test was carried out using OriginPro 2020 (OriginLab Corp., USA); a statistical significance was reported for *p* < 0.05. The fatty acids, pigments, and biological activity datasets were analyzed using principal component analysis (PCA). Before the PCA, the data underwent centering and normalization. The clustered image mapping (CIM) was conducted using the PCA results. The Euclidean distance classifier and Ward's clustering method were used for CIM analysis.

## 3 Results and discussion

### 3.1 Supercritical CO_2_ extraction study

**Fig. 1:**
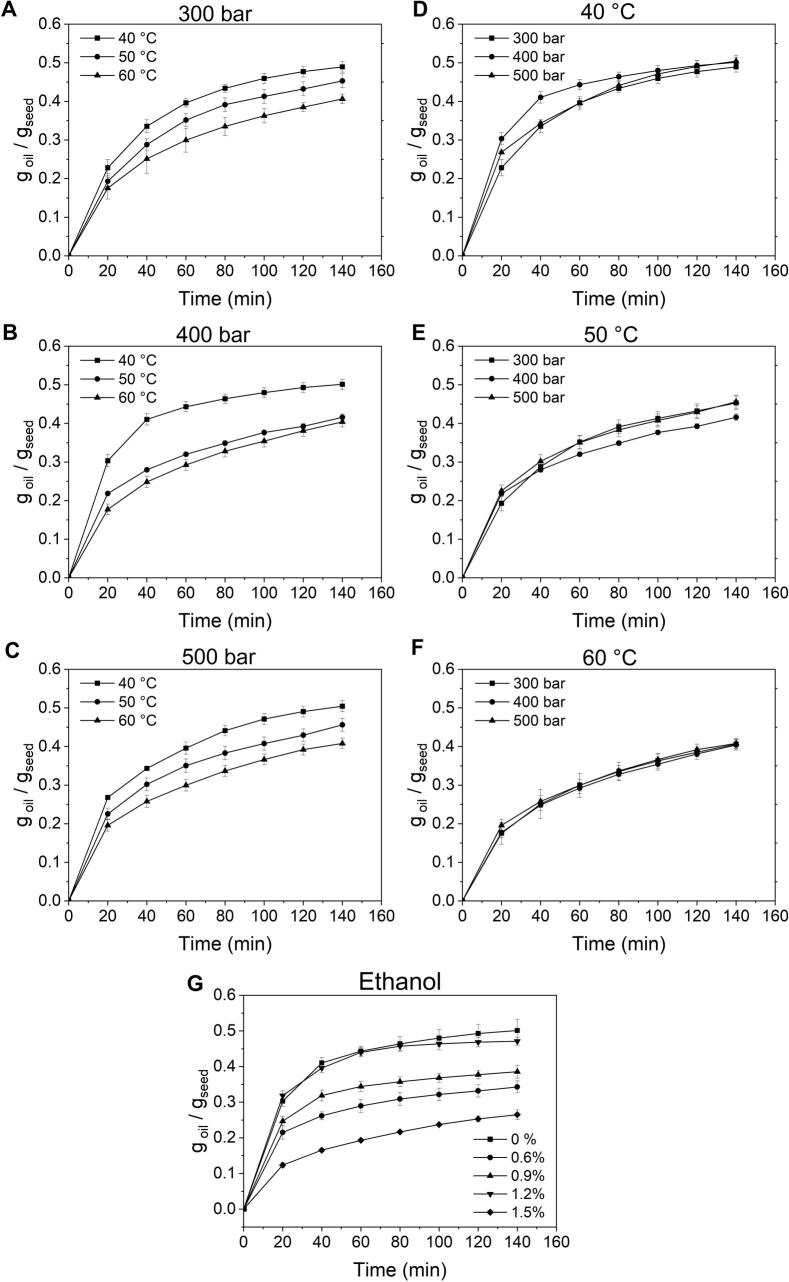
Influence of temperature (**A-C**), pressure (**D—F**), and ethanol addition (**G**) on the scCO_2_ extraction yield of hemp seed oil.

The impact of three extraction conditions (temperature, pressure, and co-solvent addition) on the yield (oil mass per hemp seed mass) was systematically studied by varying one operating parameter at a time (Table 1). The data are presented as (total) oil extraction curves (Fig. 1). The total oil yields at the end of the extraction (up to 0.5 g oil/g hemp seeds) are significantly higher than those presented in the literature (Aladić et al., 2015; C Da Porto, Decorti, & Tubaro, 2012; Carla Da Porto et al., 2015; C Da Porto, Voinovich, et al., 2012; Devi & Khanam, 2019b). The higher yields in this work are attributed to using de-hulled hemp seeds for extraction, unlike the majority of prior research which employed whole hemp seeds.. Grijó et al. (2019) also extracted de-hulled hemp seeds at pressures of 300 and 400 bar and temperatures of 40 and 60 °C. However, the highest oil yield (achieved under the conditions of 400 bar and 60 °C) was only 0.43 g/g.

**Table 1:** Experimental design matrix for studying the influence of the processing parameters on the scCO_2_ extraction of hemp seed oil.

Experiment	Pressure	Temperature	CO2 flow rate	Ethanol
	[bar]	[°C]	[g/min]	[% of CO_2_ flow rate]
E1	300	40	12.9	–
E2	300	50	12.9	–
E3	300	60	12.9	–
E4	400	40	12.9	–
E5	400	50	12.9	–
E6	400	60	12.9	–
E7	500	40	12.9	–
E8	500	50	12.9	–
E9	500	60	12.9	–
E10	400	40	12.9	0.6
E11	400	40	12.9	0.9
E12	400	40	12.9	1.2
E13	400	40	12.9	1.5

The impact of temperature (40–60 °C) clearly demonstrated that the highest hemp seed oil yields were achieved at 40 °C, regardless of pressure (Fig. 1A-C). This trend may be attributed to a decrease in scCO_2_ density with the increase in temperature (McHugh & Krukonis, 2013). Although temperatures as high as 80 °C have been reported in the literature to enhance extraction yields (Tomita et al., 2013), this study limited the temperature range to 40–60 °C. Although higher temperatures might enhance efficiency, they also pose issues regarding the thermal stability of sensitive compounds. This includes the potential for artifact formation, such as cis-trans isomerization, oxidation of PUFAs, and the degradation of tocopherols and carotenoids.

The influence of pressure was evaluated over values varying from 300 to 500 bar (Fig. 1D-F). Lower pressures (<300 bar) were not studied, as they have been shown in the literature to lead to significantly lower hemp seed oil yields (Devi & Khanam, 2019a; Tomita et al., 2013). Conversely, earlier research focused solely on pressures reaching 400 bar. For this reason, we decided to explore pressures exceeding this benchmark. However, elevating the set pressure did not result in a higher oil yield. Generally, as pressure increases, the density of the supercritical fluid also rises, thereby improving the solubility of target compounds. However, high pressures can also limit the diffusion of scCO_2_ through the material and potentially reduce the recovery of the target molecules (McHugh & Krukonis, 2013). Due to the lack of significant advantages in using higher pressures for extracting hemp seed oil and the operational and technical difficulties they introduce, we determined that an optimal pressure range for this process is 300–400 bar. A notable drawback of scCO_2_ is its low polarity.

Adding polar modifiers (co-solvents) tunes the supercritical fluid polarity, allowing for enhanced extraction of more polar molecules (L. Baldino, Scognamiglio, & Reverchon, 2020). In this work, ethanol was added as a co-solvent. The percentage of ethanol was increased from 0.6 % to 1.5 % (of total CO_2_ flow rate) while the pressure (400 bar) and temperature (40 °C) were kept constant. It was noticed that from 0.6 % to 1.2 %, the hemp seed oil extraction increased gradually. After that, a lower oil amount was obtained at 1.5 % (Fig. 1G). Several authors reported similar effects regarding the impact of the co-solvent flow rate on the oil yield (Devi & Khanam, 2019b).

Overall, regardless of the modifier's percentage, the yields obtained by adding ethanol did not surpass the yield achieved under the same pressure and temperature settings in the absence of the co-solvent (Fig. 1G). Taking into account these findings, alongside the extra expenses involved in using ethanol, its necessary removal at the process's completion, and the operational demand for an additional pump to introduce it, we determined that adding ethanol does not enhance the scCO2 extraction of hemp seed oil. Nevertheless, this conclusion is strictly based on the mass of the oil extracted. It still needs to be individually evaluated whether ethanol or varying pressure and temperature conditions affect the chemical composition or, more critically, the bioactivity of the oils.

### 3.2 Phytochemical study

#### 3.2.1 Fatty acid composition of hemp seed oil

**Fig. 2:**
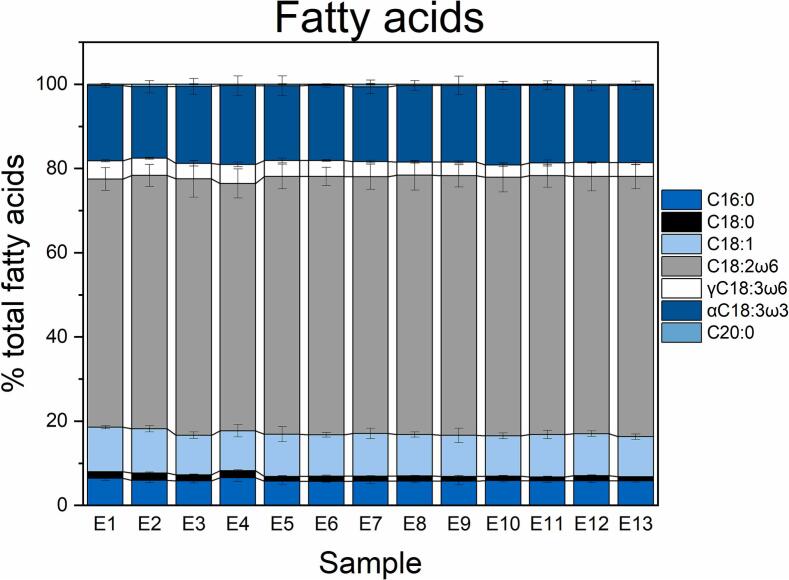
Composition of the hemp seed oils obtained under different scCO_2_ conditions. Sample code as in Table 1. C16:0, palmitic acid; C18:0, stearic acid, C18:1, oleic acid, C18:2ω6, linoleic acid; γC18:3ω6, γ-linolenic acid; αC18:3ω3, α-linolenic acid, C20:0, arachidic acid. Data are presented as average ± standard deviation of three repeated measurements.

The nutritional value of hemp seed oil is correlated mainly to its fatty acid profile. The fatty acid analysis of the 13 oils obtained under different scCO_2_ operating conditions (Fig. 2) revealed a profile typical of hemp seeds, comprising saturated fatty acids (SFAs, i.e., stearic acid, arachidic acid, palmitic acid), monounsaturated fatty acids (MUFAs, i.e., oleic acid), and PUFAs (i.e., linoleic acid, α- and γ-linolenic acids) (N. Baldino et al., 2022; Devi & Khanam, 2019a; Tura, Mandrioli, Valli, & Toschi, 2023). Of these, linoleic acid (C18:2ω6) was the most abundant derivative (57.37–61.76 % of the total fatty acids). α-Linolenic acid (αC18:3ω3, 16.99–18.99 %), oleic acid (C18:1, 9.43–11.55 %), and palmitic acid (C16:0, 5.68–6.42 %) were also present in considerable amounts. Stearic acid (C18:0), γ-linolenic acid (γC18:3ω6), and arachidic acid (C20:0) showed relatively lower values. Overall, changes in the scCO_2_ operating conditions did not visibly affect the qualitative or quantitative profile of the individual fatty acids in the obtained samples.

**Fig. 3:**
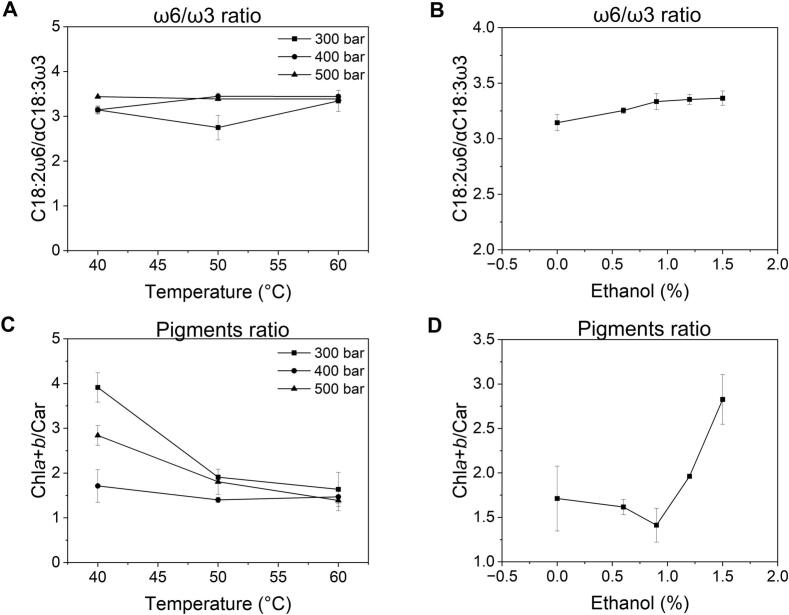
Effect of temperature, pressure, and co-solvent on the ω6/ω3 ratio (**A** and **B**) and chlorophylls/carotenoids ratio (**C** and **D**) in the hemp seed oils. C18:2ω6, linoleic acid; αC18:3ω3, γ-linolenic acid; Chl*a* + *b*, chlorophylls *a* and *b*; Car, carotenoids. Data are presented as average ± standard deviation of three repeated measurements.

Next, the impact of temperature, pressure, and co-solvent addition on the ratio between linoleic acid (C18:2ω6) and α-linolenic acid (αC18:3ω3) in the extracted oils was analyzed (Fig. 3A, B). A perfectly balanced diet should have an ω6/ω3 ratio between 2.5:1 and 5:1, as this range allegedly lowers LDL-cholesterol, improves cardiovascular function, and strengthens the immune system (Devi & Khanam, 2019b; Tomita et al., 2013). According to previous studies, a high-quality hemp seed oil should maintain a C18:2ω6/αC18:3ω3 ratio of around 3:1 (Aiello et al., 2020; Aladić et al., 2014). Except for the oil obtained at 300 bar and 50 °C (sample E2, ratio 2.75:1), all the other oils had a C18:2ω6/ αC18:3ω3 ratio between 3.14:1 and 3.45:1. Interestingly, the increase in the ethanol percentage (Fig. 3B) contributed to a slight increase in the C18:2ω6/ αC18:3ω3 ratio from 3.14:1 (at 0 % ethanol) to 3.37:1 (at 1.5 % ethanol). In conclusion, within the ranges of extraction process parameters (pressures, temperatures, and co-solvent flow rates) examined in this study, the scCO_2_-extracted hemp seed oils maintain a physiologically balanced ratio of ω6 and ω3 fatty acids.

#### 3.2.2 Tocopherol content

**Fig. 4:**
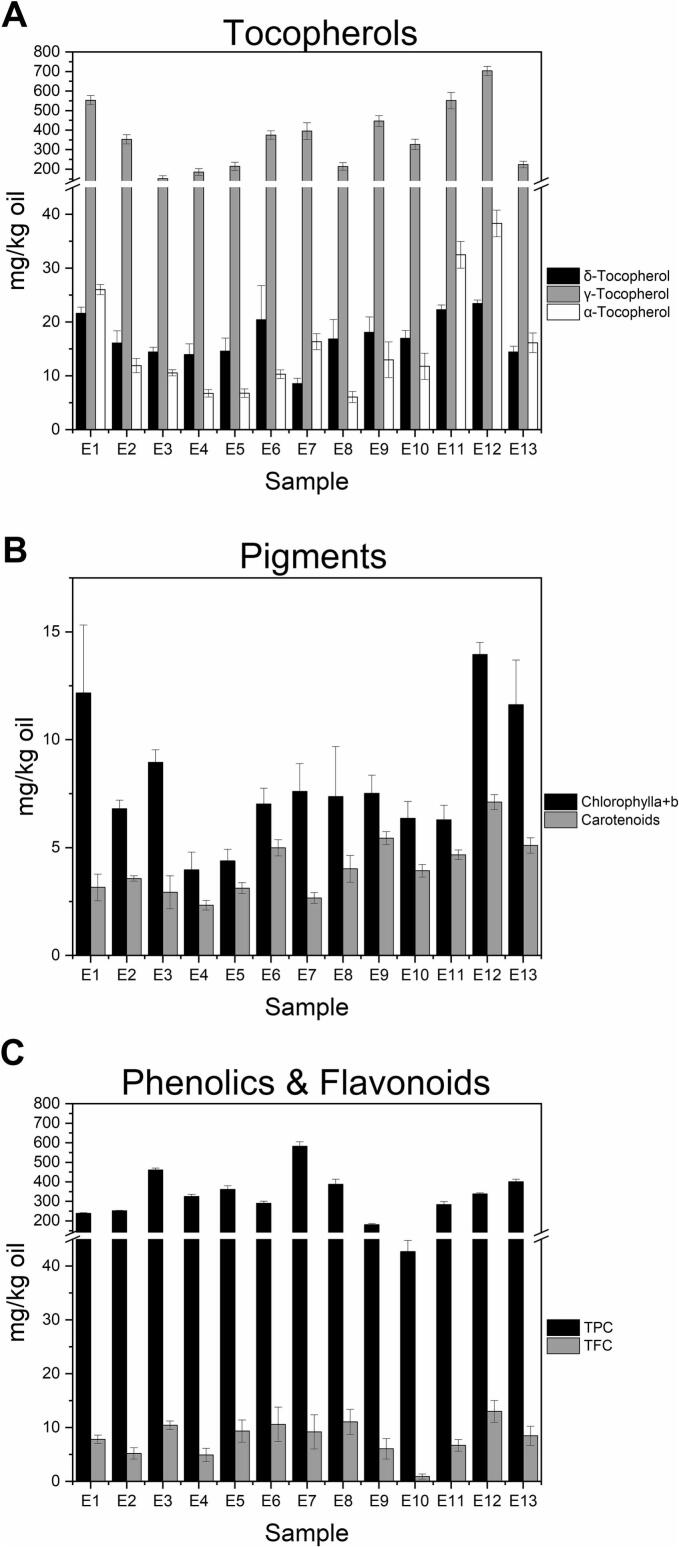
The content of tocopherols (**A**), chlorophylls *a* *+* *b* and carotenoids (**B**), phenolics, and flavonoids (**C**) in the hemp seed oils obtained under different scCO_2_ extraction conditions. Sample code as in Table 1. Data are presented as average ± standard deviation of three repeated measurements.

The scCO_2_ hemp seed oils were then analyzed for the content of several minor bio-functional compounds, such as tocopherols, chlorophylls, carotenoids, phenolics, and flavonoids (Fig. 4). Tocopherols are lipophilic antioxidants, which inhibit the oxidation of oils rich in PUFAs (Tura et al., 2023). Apparently, tocopherols found in hemp oils may help lower the risk of heart disease, delays in skin aging, and cancer (Aladić et al., 2016). The literature generally shows a wide variability in tocopherol content in hemp seed oils, from 4.30 to 850.00 mg/kg (Devi & Khanam, 2019a; Izzo et al., 2020; Teh & Birch, 2013). Among tocopherols, γ-tocopherol is the most abundant derivative in hemp seed oils, followed by α- and δ-tocopherols (Izzo et al., 2020). In this work, the γ-tocopherol content in the 13 hemp seed oils ranged from 151.12 to 703.45 mg/kg. The α- and δ-tocopherols' levels (Fig. 4A) were considerably lower (6.06 to 38.32 mg/kg and 8.57 to 23.44 mg/kg, respectively). While there was no consistent pattern found in tocopherol content related to extraction pressure and temperature, introducing ethanol markedly improved their extraction efficiency. This improvement is due to the polar aromatic hydroxyl group in tocopherols, which enhances their solubility within scCO_2_-ethanol mixtures rather than in pure scCO_2_. Nevertheless, when ethanol concentration is excessive, the long non-polar aliphatic chains of tocopherols may restrict additional solubility improvements, which could account for the decrease in efficiency at the highest ethanol concentrations. This phenomenon highlights the amphiphilic nature of tocopherols, making them partially compatible with both polar and non-polar environments.

#### 3.2.3 Total pigment content

Carotenoids and chlorophylls represent the primary pigments in hemp oils (Aiello et al., 2020). (Aiello et al., 2020). In addition to their potential use as natural food colorants, carotenoids are believed to possess antioxidant properties and may provide benefits in preventing age-related degenerative diseases, atherosclerosis, and cancer (Aladić et al., 2014). Natural chlorophylls are quite unstable, often breaking down when subjected to light, acidic conditions, or oxygen. Consequently, employing scCO_2_ is believed to yield superior quality oil by avoiding the excessive heating that might lead to chlorophylls' degradation. (Aladić et al., 2016).

In addition, the pigment levels are indicative of the type of material used: oils obtained from de-hulled hemp seeds, as in the current work, typically have a light green color, reflecting its lower pigment content (chlorophylls: 0.41–4.81 mg/kg oil; carotenoids: 0.18–1.73 mg/kg oil) (Izzo et al., 2020). In contrast, oils extracted from whole (un-hulled) seeds would exhibit a darker green color, corresponding to a higher pigment content (chlorophylls: 130.40–456.30 mg/kg oil; carotenoids: 2.53–132.67 mg/kg oil) (Aiello et al., 2020; Aladić et al., 2015; Devi & Khanam, 2019a; Tura et al., 2023). In this study, the chlorophylls in the hemp seed oils obtained under various scCO_2_ operating conditions, measured as the sum of chlorophyll *a* and *b*, ranged from 3.97 to 13.95 mg/kg, while the carotenoid levels varied from 2.33 to 7.11 mg/kg (Fig. 4B). While pressure and temperature had minimal effects on chlorophyll and carotenoid content, ethanol addition improved their extractability. The increasing polarity of the supercritical fluid achieved by ethanol addition enhanced pigment solubility due to the presence of oxygenated functional groups in their structures. However, like tocopherols, these pigments also have long aliphatic chains, which may hinder solubility in highly polar systems, likely contributing to the decline in recovery at 1.5 % ethanol. Excessive ethanol addition can disrupt the solvating power of scCO_2_ by reducing its density, which may lower its overall extraction capacity for semi-polar compounds.

Excessively high amounts of chlorophylls are known to enhance the photo-oxidation of oils (Aiello et al., 2020). Thus, the impact of temperature, pressure, and co-solvent addition on the ratio between the green (chlorophylls) and yellow (carotenoids) fractions was next analyzed (Fig. 3C, D). While pressure did not appear to affect the pigment ratio, temperature showed an influence. The chlorophyll/carotenoid ratio was lower at higher temperatures (50–60 °C), indicating a possible thermal degradation of carotenoids (Fig. 3C). On the other hand, the addition of ethanol had a variable effect; the chlorophyll/carotenoid ratio decreased as the percentage was varied from 0 to 1.2 %, but beyond this point, the ratio between the two pigments increased (Fig. 3D). Overall, the chlorophyll/carotenoid ratio of the 13 hemp seed oil obtained under different scCO_2_ operating conditions ranged from 1.4 to 3.9.

#### 3.2.4 Total phenolic and flavonoid content

The total flavonoid and phenolic content of the 13 hemp seed oils is presented in Fig. 4C. The phenolic content varied from 42.68 to 581.70 mg GAE/kg, whereas the flavonoid content ranged between 0.89 and 13.00 mg RE/kg. The temperature did not clearly affect the extraction of these two categories of phytochemicals; however, the pressure appeared to influence their recovery. Their recovery increased with pressure at lower temperatures (40 and 50 °C), likely due to increased scCO_2_ density enhancing solubilization. However, at 60 °C, higher pressure resulted in decreased concentrations, possibly due to thermal degradation. Across all temperatures, ethanol addition improved phenolic and flavonoid extraction, consistent with their strong polarity and poor solubility in pure scCO_2_. Moreover, hydrogen bonding and π–π interactions between ethanol and these compounds may enhance their solubility and transport into the supercritical phase. Limited literature is available on the total phenolic content of hemp seed oils, and virtually no studies have reported on their flavonoid content. The total phenolic content of oils extracted with hexane, liquid CO_2_, and sCO_2_ ranged from 33.59 to 47.18 mg GAE/kg (Aiello et al., 2020). In other studies, phenolics in commercial hemp seed oils varied from 16.75 to 186.78 mg GAE/kg (Izzo et al., 2020; Tura et al., 2022).

### 3.3 Biological study

#### 3.3.1 Antioxidant activity

Although hemp seed oil has been attributed to numerous health benefits (Leonard, Zhang, Ying, & Fang, 2020), there is a lack of solid scientific evidence to support these claims. Antioxidant activities, especially their ability to neutralize radicals, play a vital role in protecting against the detrimental impacts of free radicals in biological systems. These reactive species can harm essential cellular components such as the nucleus and membranes, including DNA, proteins, carbohydrates, and lipids (Smeriglio et al., 2016). The DPPH and ABTS radical scavenging properties of the 13 scCO_2_ hemp seed oils obtained under different process conditions are presented in Table 2. The scavenging activity ranged from 397.58 to 522.47 mg TE/100 g in the ABTS assay and from 160.19 to 251.87 mg TE/100 g in the DPPH assay. The radical scavenging effects of hemp seed oils were tackled in previous studies (Aloo, Kwame, & Oh, 2023; Carla Da Porto et al., 2015; Gulcin et al., 2024; Hong et al., 2015; Irakli et al., 2019; Kitamura et al., 2020; Lakatos, Apori, Dunne, & Tian, 2022; Manosroi et al., 2019; Smeriglio et al., 2016; Vitorović et al., 2021; Yu et al., 2005). The DPPH data from these studies cannot always be directly compared due to the different methods of expressing the activity (e.g., % inhibition, tocopherol equivalents per mL oil, inhibitory concentration 50/IC_50_, etc.). For the ABTS assay, the radical scavenging activity of various hemp seed oil samples was previously shown to vary between 174.00 and 285.33 mg TE/100 g (Smeriglio et al., 2016; Yu et al., 2005).

**Table 2:** Antioxidant activity of the hemp seed oils obtained under different scCO_2_ extraction conditions.

Exp.	Conditions	DPPH	ABTS	CUPRAC	FRAP	MCA	PBD
		mg TE/100 g				mg EDTAE/100 g	mmol TE/100 g
E1	300 bar/40 °C	250.79 ± 24.91^a^	522.47 ± 25.07^a^	1036.83 ± 6.04^a^	407.86 ± 11.85^a^	595.44 ± 12.61^a^	16.19 ± 1.85^a^
E2	300 bar/50 °C	183.65 ± 5.69^b^	397.58 ± 2.79^b^	1280.24 ± 19.21^b^	494.87 ± 20.85^b^	670.05 ± 7.04^b^	16.91 ± 0.32^a,b^
E3	300 bar/60 °C	192.49 ± 9.41^c^	418.07 ± 9.41^c,d^	1621.10 ± 17.16^c^	564.34 ± 4.27^c^	766.11 ± 24.42^c^	17.50 ± 0.75^b^
E4	400 bar/40 °C	160.19 ± 13.13^d^	415.58 ± 4.47^c,d^	1417.88 ± 37.13^d^	467.93 ± 12.35^d^	893.61 ± 7.54^d^	19.05 ± 0.37^c^
E5	400 bar/50 °C	192.72 ± 5.12^c^	406.47 ± 6.43^b,c^	1407.66 ± 8.79	505.18 ± 46.69^b^	635.07 ± 15.95^e^	20.53 ± 1.42^d^
E6	400 bar/60 °C	188.00 ± 21.31^b^	422.98 ± 8.31^d^	1143.28 ± 86.84^a,b,e^	429.24 ± 14.16^a^	701.02 ± 3.06^f^	14.93 ± 0.43^e^
E7	500 bar/40 °C	163.00 ± 7.60^d^	429.74 ± 7.07^d^	2443.95 ± 15.21^f^	479.82 ± 27.74^b,d^	983.01 ± 19.43^g^	25.72 ± 0.45^f^
E8	500 bar/50 °C	192.90 ± 25.96^c^	450.66 ± 18.59^e^	1184.81 ± 12.50^e^	462.37 ± 1.69^b,d^	682.26 ± 20.18^b,f^	15.44 ± 0.56^e^
E9	500 bar/60 °C	189.73 ± 9.23^b,c^	415.58 ± 10.18^c^	1087.50 ± 45.53^g^	429.02 ± 6.11^a^	698.54 ± 59.27^b,f^	14.69 ± 0.35^e^
E10	400 bar/40 °C + 0.6 % ethanol	200.66 ± 17.81^c^	440.98 ± 37.91^e^	969.09 ± 32.56^a^	405.06 ± 5.08^a^	519.69 ± 25.13^h^	13.78 ± 0.67^f^
E11	400 bar/40 °C + 0.9 % ethanol	169.86 ± 5.66^d^	427.89 ± 5.93^d^	1277.15 ± 27.83^e^	477.02 ± 11.42^b,d^	518.26 ± 17.54^h^	22.56 ± 6.50^g^
E12	400 bar/40 °C + 1.2 % ethanol	199.34 ± 38.71^c^	438.42 ± 19.23^d^	1171.91 ± 13.92^e^	454.96 ± 3.84^d^	472.28 ± 24.88^i^	15.20 ± 0.91^e^
E13	400 bar/40 °C + 1.5 % ethanol	251.87 ± 11.77^a^	494.63 ± 15.84^a^	959.54 ± 34.42^a^	357.51 ± 9.52^e^	878.26 ± 5.16^d^	14.45 ± 1.18^e^

ABTS, 2,2′-azino-bis(3-ethylbenzothiazoline) 6-sulfonic acid; CUPRAC, cupric ion reducing antioxidant capacity; DPPH, 1,1-diphenyl-2-picrylhydrazyl; EDTAE, ethylenediaminetetraacetic acid equivalents; FRAP, ferric ion reducing antioxidant power; MCA, metal chelating activity PBD, phosphomolybdenum assay; TE, Trolox equivalents Data are presented as average ± standard deviation of three repeated measurements. Values within columns with different superscript letters indicate significant differences (*p* < 0.05).

The reducing power of a molecule can also indicate its putative antioxidant properties. Antioxidant compounds in the samples facilitate the conversion of ferric and cupric ions to their ferrous and cuprous forms. In this work, the reducing capacity of hemp seed oils ranged from 357.51 to 564.34 mg TE/100 g in FRAP assay and 969.09 to 2443.95 mg TE/100 g oil in CUPRAC assay. Previously, the FRAP of a cold-pressed hemp seed oil sample was estimated at 991.31 mg TE/100 g (Aloo et al., 2023; Irakli et al., 2019; Smeriglio et al., 2016). In addition, transition metals can act as catalysts, promoting the formation of free radicals that start oxidative chain reactions (Smeriglio et al., 2016). In this work, the chelating activity of the scCO_2_ hemp seed oils ranged from 472.28 to 983.01 mg EDTAE/100 g. A previous study reported a significantly lower chelating activity (46.23 mg EDTAE/100 g) for a cold-pressed hemp oil sample (Smeriglio et al., 2016). Lastly, the total antioxidant activity of the 13 hemp seed oils was measured using the PBD assay, with values varying from 13.78 to 25.72 mmol TE/100 g (Table 2). No comparison with the literature was possible for this test.

When examining the impact of pressure and temperature on the antioxidant activity of hemp seed oils, it was noted that the scavenging activities of the extracts obtained at 40 °C decreased as the extraction pressure increased. However, the oils recovered at higher temperatures (50 and 60 °C) showed nearly the same scavenging activity regardless of the pressure used for their extraction. In contrast, the reducing and chelating abilities of extracts obtained at 40 °C were enhanced as the extraction pressure increased. Thus, the highest ABTS and DPPH activity was seen in oils produced at 40 °C and 300 bar, while the oil at 40 °C and 500 bar showed superior activity in the other four antioxidant tests. The effect of ethanol addition varied depending on the type of assay. In radical scavenging tests, higher co-solvent flow rates generally led to an increased effect. However, the opposite trend was recognized for the reducing assays, where higher flow rates led to lower antioxidant activity. Moreover, there was no discernible pattern in the results of the MCA and PBD assays. In general, the various impacts of the scCO2 operating conditions on the scavenging, reducing, and chelating assays can be linked to the participation of different types of molecules in these activities. Nonetheless, pinpointing the specific compounds from the prior analysis that most significantly influenced the observed outcomes remains challenging.

#### 3.3.2 Enzyme inhibition

To date, hemp seed oils have been scarcely included in enzyme inhibition screening platforms (Aloo et al., 2023; Gulcin et al., 2024; Haddou et al., 2023; Manosroi et al., 2019; Sangkanu et al., 2023). The findings on the enzyme inhibitory effects of hemp seed oils are detailed in Table 3. The AChE inhibition displayed by the oils was evaluated at 89.95–102.49 mg GALAE/100 g, while the BChE inhibition was 4 to 4.5 times higher (389.86–478.09 mg GALAE/100 g). In a previous study (Gulcin et al., 2024), hemp seed oil exhibited an IC_50_ value against AChE of 28.00 μg/mL. Tyrosinase inhibition changed from 1770.83 to 2794.88 mg KAE/100 g. Previously, a hemp seed extract displayed an IC_50_ value against tyrosinase of 0.07 mg/mL (Manosroi et al., 2019). Compared to the anti-amylase activity (54.64–70.10 mmol ACAE/100 g), the glucosidase inhibition was around 5 to 5.5 times higher (301.16–355.77 mmol ACAE/g). In a previous study (Gulcin et al., 2024), hemp seed oil showed an IC_50_ value of 545.66 μg/mL against amylase. In contrast, in another work, IC_50_ values between 19.33 and 23.53 μg/mL were reported for hemp seeds extracted with different solvents (Haddou et al., 2023). Concerning glucosidase, some reports (Haddou et al., 2023; Sangkanu et al., 2023) presented IC_50_ values between 33.23 and 94.09 μg/mL. In conclusion, the oils demonstrated a more potent inhibitory effect against BChE and glucosidase than against AChE and amylase.

**Table 3:** Enzyme inhibition of the hemp seed oils obtained under different scCO_2_ extraction conditions.

Exp.	Conditions	AChE	BChE	Tyrosinase	Amylase	Glucosidase
		mg GALAE/100 g		mg KAE/100 g	mmol ACAE/100 g	
E1	300 bar/40 °C	101.84 ± 0.31^a^	389.86 ± 6.00^a^	1781.23 ± 163.50^a^	62.88 ± 0.95^a^	346.05 ± 21.09^a^
E2	300 bar/50 °C	100.76 ± 0.56^a^	478.09 ± 32.85^b^	2794.88 ± 52.55^b^	60.81 ± 0.58^b^	355.77 ± 5.92^a^
E3	300 bar/60 °C	102.49 ± 0.17^a^	424.85 ± 58.49^c^	1991.92 ± 137.83	64.85 ± 2.18^c^	336.60 ± 9.07^b^
E4	400 bar/40 °C	99.44 ± 0.69^b^	428.35 ± 3.84^c^	1924.29 ± 248.34^c^	70.10 ± 1.66^d^	316.12 ± 12.68^c^
E5	400 bar/50 °C	99.84 ± 0.16^b^	450.41 ± 28.38^b,c^	1770.83 ± 120.44^a^	59.83 ± 1.42^b^	349.83 ± 6.83^a^
E6	400 bar/60 °C	89.95 ± 1.04^c^	461.16 ± 41.37^b,c^	2010.91 ± 134.39^c^	65.12 ± 1.47^c^	342.09 ± 3.46^a^
E7	500 bar/40 °C	99.40 ± 0.10^b^	451.37 ± 10.02^b,c^	2435.93 ± 236.44^c^	65.40 ± 1.10^c^	301.16 ± 35.61^c^
E8	500 bar/50 °C	98.88 ± 0.48^b^	444.58 ± 31.02^b,c^	1778.63 ± 85.00^c^	69.49 ± 0.63^d^	341.83 ± 14.94^a^
E9	500 bar/60 °C	98.31 ± 3.62^b^	439.71 ± 38.79^b,c^	2164.63 ± 125.12^c^	58.68 ± 0.68^b^	354.31 ± 9.45^a^
E10	400 bar/40 °C + 0.6 % ethanol	102.02 ± 0.52^a^	475.35 ± 18.67^b^	1836.37 ± 162.32^a^	63.89 ± 1.94^a^	337.28 ± 11.40^a^
E11	400 bar/40 °C + 0.9 % ethanol	98.94 ± 4.09^a,b^	421.85 ± 89.17^c^	1991.14 ± 150.82^a^	62.20 ± 1.90^b^	331.54 ± 10.41^a^
E12	400 bar/40 °C + 1.2 % ethanol	97.51 ± 1.19^b^	437.41 ± 12.62^c^	1886.83 ± 112.86^a^	54.64 ± 0.40^d^	349.25 ± 27.27^a^
E13	400 bar/40 °C + 1.5 % ethanol	98.65 ± 0.46^b^	418.24 ± 26.46^a,c^	2211.45 ± 84.41^c^	61.37 ± 0.38^b^	349.38 ± 15.55^a^

ACAE, acarbose equivalents; AChE, acetylcholinesterase; AMYL, amylase; BChE, butyrylcholinesterase; GALAE, galanthamine equivalents; GLC, glucosidase; KAE, kojic acid equivalents; TYR, tyrosinase. Data are presented as average ± standard deviation of three repeated measurements. Values within columns with different superscript letters indicate significant differences (*p* < 0.05).

Nevertheless, the enzyme inhibition of the oils obtained under different scCO_2_ extraction conditions showed minimal activity differences between the samples. As a result, no clear conclusions about the impact of pressure, temperature, or ethanol addition on the enzyme inhibitory properties of hemp seed oils could be drawn. The unclear relationship between enzyme inhibition and extraction parameters is likely due to a combination of non-linear trends in phenolic/flavonoid recovery, variability in the bioactivity of individual compounds, and possible synergistic or matrix effects not captured by phytochemical analyses performed in this study. Nevertheless, this is the first study to assess the enzyme inhibitory potential of scCO_2_-extracted hemp seed oils.

### 3.4 Exploratory multivariate analysis study

In this section, we examined the results from phytochemical and biological studies utilizing PCA and CIM to assess the variations in hemp seed oils extracted via different scCO_2_ conditions. Initially, we calculated the eigenvalues and the percentage of variances for datasets of fatty acids and pigments as shown in Table S1. Our findings indicated that the first two principal components (PCs) accounted for 87 % of the total variation in fatty acids and 82 % in pigments (Table S1).

**Fig. 5:**
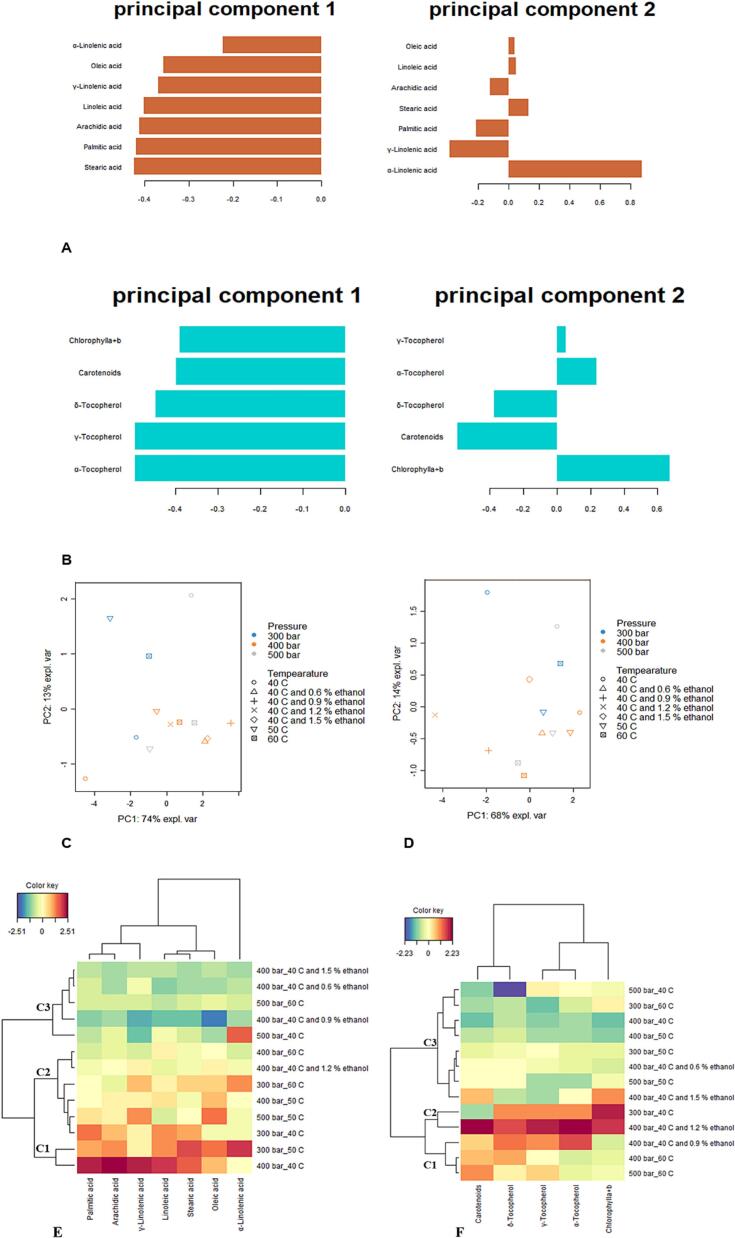
Exploratory multivariate analysis on fatty acid and pigment datasets. Contribution of fatty acids on the first two components of the principal component analysis (PCA) (**A**). Contribution of pigments on the first two components of PCA **(B**). Scatter plots of PCA for fatty acids (**C**) and pigments (**D**). Clustered image maps showing the global overview of the fatty acids (**E**) and pigments (**F**) contrasts among samples. (Blue color: low content. Red color: high content). (For interpretation of the references to color in this figure legend, the reader is referred to the web version of this article.)

The contribution of each compound to these two PCs is illustrated in Fig. 5A, B. For fatty acids, PC1 is primarily influenced by stearic, palmitic, and arachidic acids, while for pigments, it is dominated by tocopherols. Thus, PC1 can represent the variation in saturated fatty acids and tocopherols. For fatty acids, PC2 showed a strong positive loading for α-linolenic acid, while for pigments, PC2 had the highest positive loading for chlorophylls and the highest negative loading for carotenoids.

Fig. 5C and D show the sample distribution on a scatter plot using PC1 vs. PC2 for fatty acids and pigments, respectively. Although the oil samples show significant variability of their fatty acid and pigment contents, no apparent clustering of extracts is evident. To understand better the shared characteristics among the samples, a CIM was generated based on the PCA results. As shown in Fig. 5E and F, the samples were grouped into three clusters for each analysis. Samples E2 (300 bar/50 °C) and E4 (400 bar/40 °C) in cluster C1 had the highest content of fatty acids (Fig. 5E). Similarly, the samples in cluster C2 (E1 at 300 bar/40 °C and E12 at 400 bar/40 °C with 1.2 % ethanol) had the highest pigment content (Fig. 5F).

**Fig. 6:**
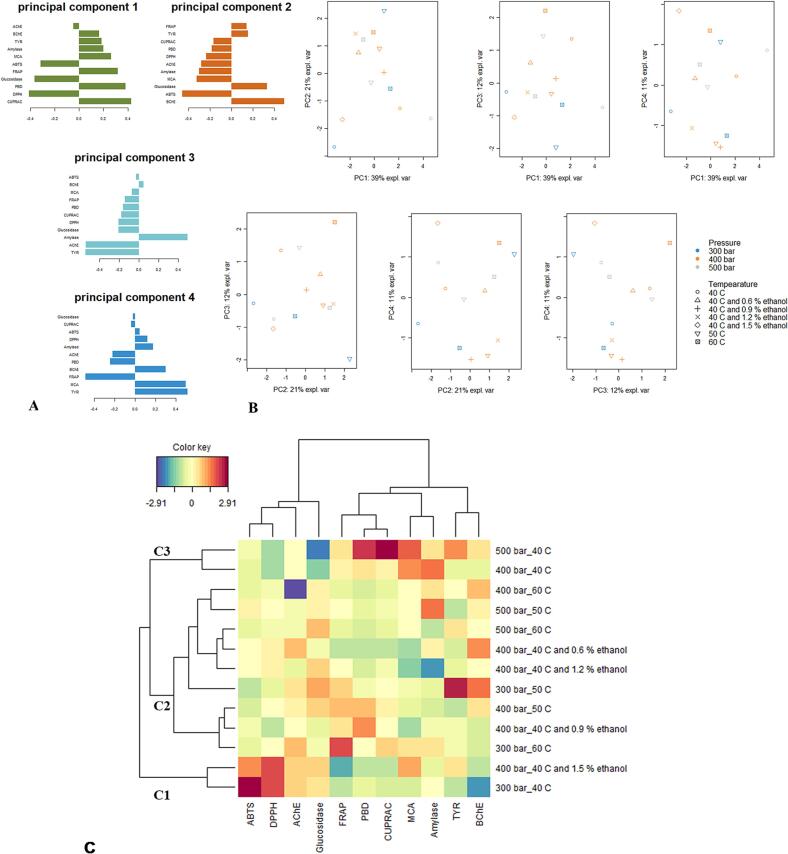
Exploratory multivariate analysis on biological activities datasets. Contribution of biological activities on the first four components of the principal component analysis (PCA) (**A**). Scatter plots of PCA (**B**). Clustered image map (**C**) showing the global overview of the biological activity contrasts among samples. (Blue color: low bioactivity. Red color: high bioactivity). (For interpretation of the references to color in this figure legend, the reader is referred to the web version of this article.)

PCA was also used to identify groups of samples with similar biological activities. After performing PCA, the variance explained by each PC was determined. The first four PCs accounted for approximately 83 % of the variation among samples, with PC1 explaining about 39 %, PC2 21 %, PC3 12 %, and PC4 11 % (Table S2). Fig. 6A highlights which bioactivities contribute most to each PC. CUPRAC, DPPH, PBD, and glucosidase were significant contributors to PC1, while ABTS and BChE were important for PC2. PC3 was mainly influenced by tyrosinase, AChE, and amylase, whereas PC4 was directed by FRAP, tyrosinase, and MCA. Fig. 6B shows clusters of samples based on their biological activity similarities. However, it was challenging to distinguish different clusters from these graphs. To aid in cluster identification, a CIM was created based on the PCA results (Fig. 6C), revealing three main clusters. Cluster C1, containing samples E1 (300 bar/40 °C) and E12 (400 bar/40 °C with 1.2 % ethanol), exhibited high ABTS and DPPH radical scavenging activity. Notably, this cluster overlaps with the pigment-rich cluster C2, indicating a possible contribution of this category of compounds to the antioxidant activity of hemp seed oils (Fig. 5E). A high MCA activity was pointed out by cluster C3, which includes samples E4 (400 bar/40 °C) and E7 (500 bar/40 °C). Overall, using exploratory multivariate analysis, it was possible to group the scCO_2_-extracted hemp seed oils in several clusters regarding their phytochemical profile and biological activity.

## 4 Conclusion

This study highlighted that scCO_2_ extraction is a promising method for obtaining high-quality oil from de-hulled hemp seeds, with tunable parameters influencing both yield and bioactivity. Among the variables tested, temperature and co-solvent addition emerged as the most influential factors, particularly in enhancing oil yield and the recovery of minor bioactive components such as tocopherols, chlorophylls, carotenoids, phenolics, and flavonoids. Notably, ethanol significantly improved the extractability of these compounds without compromising oil quality. The fatty acid profile remained remarkably stable across all processing conditions, maintaining the ω6/ω3 ratio typical of hemp seed oil. This suggests that scCO_2_ extraction is capable of preserving essential lipid constituents, rendering it highly suitable for applications in the nutraceutical sector. Although antioxidant and enzyme inhibitory activities were observed across all extracts, the variation in these activities did not exhibit a straightforward relationship with the processing parameters. This lack of linearity suggests that compound-specific or synergistic effects likely play a critical role. Therefore, the findings highlight the need for tailoring scCO_2_ extraction conditions according to the desired functionality. For example, if maximizing tocopherol and pigment content is the target, conditions such as 400 bar, 40 °C, and 1.2 % ethanol are optimal. Conversely, for enhancing radical scavenging or enzyme inhibitory activities, other pressure-temperature combinations may be more suitable (e.g., 300 bar and 50 °C). Nonetheless, the continued utilization of multivariate analysis methodologies may assist in the precise adjustment of scCO_2_ processing parameters, thereby facilitating the attainment of more consistent and repeatable bio-functional characteristics in hemp seed oils.

## CRediT authorship contribution statement

**Prajakta Vishwasrao:** Writing – review & editing, Investigation, Formal analysis. **Gokhan Zengin:** Writing – review & editing, Investigation, Formal analysis, Data curation. **Kouadio Ibrahime Sinan:** Writing – original draft, Investigation, Data curation. **Mirjana Minceva:** Writing – review & editing, Funding acquisition. **Simon Vlad Luca:** Writing – original draft, Validation, Supervision, Project administration, Methodology, Data curation, Conceptualization.

## Declaration of competing interest

The authors declare that they have no known competing financial interests or personal relationships that could have appeared to influence the work reported in this paper.

## Data Availability

Data will be made available on request.

## Appendix A Supplementary data

Supplementary material Image 1
