# Supplementary material for: Supercritical CO2 extraction of hemp seeds: A multivariate perspective on the influence of processing parameters on oil composition, antioxidant activity, and enzyme inhibition
Source: Food Chem X. 2025 Nov 16;32:103296. doi: 10.1016/j.fochx.2025.103296 (PMC12670116; doi:10.1016/j.fochx.2025.103296)
Supplement: Supplementary file 1 — Supplementary material [file mmc1.docx]

**SUPPLEMENTARY MATERIAL for**

**Supercritical CO_2_ extraction of hemp seeds: A multivariate perspective on the influence of processing parameters on oil composition, antioxidant activity, and enzyme inhibition**

Prajakta Vishwasrao^a^, Gokhan Zengin^b^, Kouadio Ibrahime Sinan^b^, Mirjana Minceva^a^, Simon Vlad Luca^a,*^

^a^ *Biothermodynamics, TUM School of Life Sciences, Technical University of Munich, 85354 Freising, Germany*

*^b^ Physiology and Biochemistry Research Laboratory, Department of Biology, Science Faculty, Selcuk University, 42130 Konya, Turkey*

*Correspondence: [vlad.luca@tum.de](mailto:vlad.luca@tum.de)


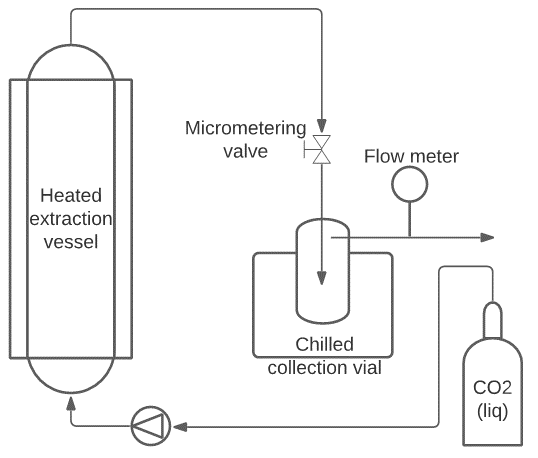


**Fig.** **S1.** Schematic representation of scCO_2_ extractor

**Table S1.** Eigenvalues and proportion of explained variance for the first four principal components concerning the phytochemical data

| **Principal components** | | **PC1** | **PC2** | **PC3** | **PC4** |
| --- | --- | --- | --- | --- | --- |
| **Fatty acids** | Eigenvalues | 5.18 | 0.93 | 0.57 | 0.15 |
|  | Proportion of explained variance | 74.01 | 13.31 | 8.23 | 2.13 |
| **Pigments** | Eigenvalues | 3.39 | 0.69 | 0.47 | 0.33 |
|  | Proportion of explained variance | 67.84 | 13.96 | 9.43 | 6.72 |

**Table S2**. Eigenvalues and proportion of explained variance for the first five principal components concerning the biological activity data

| **Principal components** | **PC1** | **PC2** | **PC3** | **PC4** | **PC5** |
| --- | --- | --- | --- | --- | --- |
| Eigenvalues | 4.27 | 2.26 | 1.29 | 1.24 | 0.78 |
| Proportion of explained variance | 38.86 | 20.52 | 11.72 | 11.30 | 11.30 |
